# Two decades of experience in explantation and graft preserving strategies following primary endovascular aneurysm repair and lessons learned

**DOI:** 10.3389/fsurg.2022.963172

**Published:** 2022-08-09

**Authors:** Sherif Sultan, Yogesh Acharya, Mohieldin Hezima, Keegan Chua Vi Long, Osama Soliman, Juan Parodi, Niamh Hynes

**Affiliations:** ^1^ Western Vascular Institute, Department of Vascular and Endovascular Surgery, University Hospital Galway, National University of Ireland, Galway, Ireland; ^2^ Galway: Department of Vascular Surgery and Endovascular Surgery, Galway Clinic, Doughiska, Royal College of Surgeons in Ireland and National University of Ireland, Galway affiliated Hospital, Galway, Ireland; ^3^ CORRIB-CURAM-Vascular Group, National University of Ireland, Galway, Ireland; ^4^ Department of Vascular Surgery and Biomedical Engineering Department, Alma mater, University of Buenos Aires, and Trinidad Hospital, Buenos Aires, Argentina; ^5^ Winston-Salem and St. Louis: Wake Forest University, Winston-Salem, North Carolina and Washington University in St. Louis, St. Louis, Missouri, United States of America

**Keywords:** endovascular procedures, complications, reintervention, explantation, graft preservation

## Abstract

**Objectives:**

We aim to scrutinize our evolving re-intervention strategies following primary endovascular aortic aneurysm repair (EVAR) - EVAR GORE SalvAge Fabric Technique (ARAFAT), aortic sac double breasting with endograft preservation, and stent-graft explantation.

**Methods:**

We performed 1,555 aortic interventions over the study period, including 910 EVARs. Factors associated with the need for reintervention and the likelihood of chronic fabric fatigue failure (CFFF) were investigated. Using conventional and innovative diagnostic modalities with Prone contrASt enHanced computed tomography Angiography (PASHA), 136 endoleaks (ELs) were identified (15 type I, 98 type II; 18 type III; 5 type IV).

**Results:**

Forty-four (4.84%) patients underwent re-intervention post-primary EVAR; 18 ARAFATs, 12 double breastings, and 14 explantations. Choice of re-intervention was based on patient fitness and mode of failure. Mean EL detection duration following primary EVAR was 53.3 ± 6.82 months, while mean time to re-intervention was 70.20 ± 6.98 months. The mean sac size before the primary EVAR and re-intervention was 6.00 ± 1.75 cm and 7.51 ± 1.94 cm, respectively. Polyester (61.40%) was the most commonly employed stent-graft material. Use of more than three modular stent-graft components (3.42 ± 1.31, *p* = 0.846); with the proximal stent-graft diameter of 31.6 ± 3.80 cm (*p* = 0.651) and the use of iliac limbs more than 17 mm (*p* = 0.364), all added together are contributing factors. We had one peri-operative mortality following explantation due to sepsis-induced multiorgan failure.

**Conclusions:**

Our re-intervention strategies matured from stent graft explantation to graft preservation with endovascular relining of the stent-graft. Graft preservation with aortic sacotomy and double breasting were used to manage concealed ELs due to aortic hygroma.

## Introduction

Endovascular aneurysm repair (EVAR) has revolutionised therapeutic tactics in managing aortic pathologies over the past three decades. EVAR has shown reduced early peri-operative morbidity and mortality compared to open surgical repair (OSR) (1). However, these advantages are lost in long-term follow-up due to stent-graft complications, including fabric and material failure (2–5).

EVAR effectiveness is determined by the aortic sac segregation from systematic pressure and sac shrinkage. Complications post-EVAR need close surveillance as aortic sac dynamics influences EVAR durability, and continuous sac expansion could result in rupture (3). Endoleaks (ELs) may thrombose, but if they persist, the consequences can be detrimental, mainly if they are high-flow and associated with continued aneurysmal sac expansion (4, 5). These complications require aggressive management with re-intervention to abolish the risk of rupture. Re-intervention could be achieved through salvage of the primary endograft *via* a graft preserving strategy or explantation as necessary. In this study, we aim to analyse our three evolving strategies of re-intervention following the primary EVAR - EVAR GORE SalvAge Fabric Technique (ARAFAT), double breasting, and explantation.

## Methods

This is a retrospective observational study performed in our tertiary vascular center from 2002 to 2020. The primary outcome is aortic related mortality. The secondary outcomes are technical success, perioperative morbidity and mortality, and overall survival probabilities.

Society for Vascular Surgery (SVS) reporting standards is used to define the outcomes (6). Technical success is defined as the periprocedural events from the initiation of the procedure to the first 24-hour. Primary technical success is the successful introduction and deployment without surgical conversion or death, type I or III ELs, or graft limb obstruction.

A re-intervention is classified as any procedure performed for subsequent aneurysm and/or primary procedure-related complications during follow-up of the primary EVAR. Factors associated with the need for re-intervention and the likelihood of chronic fabric fatigue failure (CFFF) were investigated amongst the re-intervention groups.

### Primary EVAR and follow-up strategy

Out of 22,349 aortic referrals to our tertiary referral centre, we performed 1,555 aortic interventions over twenty years. Amongst them, 910 were EVAR ± iliac branch device (IBD), and 96 were thoracic endovascular aortic repair (TEVAR)/branched endovascular aortic repair (BEVAR).

All our patients were followed-up with duplex ultrasonography (DUS) and plain film abdomen every six months. Patient with continuous sac expansion without detection of EL through conventional supine computed tomography angiography (CTA) underwent a Prone contrASt enHanced computed tomography Angiography (PASHA), a multiphase time-resolved four-phase positional (prone) CTA protocol for detection and classification of concealed ELs (7). The PASHA protocol enhanced the degree of contrast infiltration into the aortic sac when microleaks were present, which helped us to plan the re-intervention strategy. PASHA diagnosed all cases of type IIIb EL that were previously classified as concealed Type V EL in the context of continuous sac enlargement (7).

**Figure 1:**
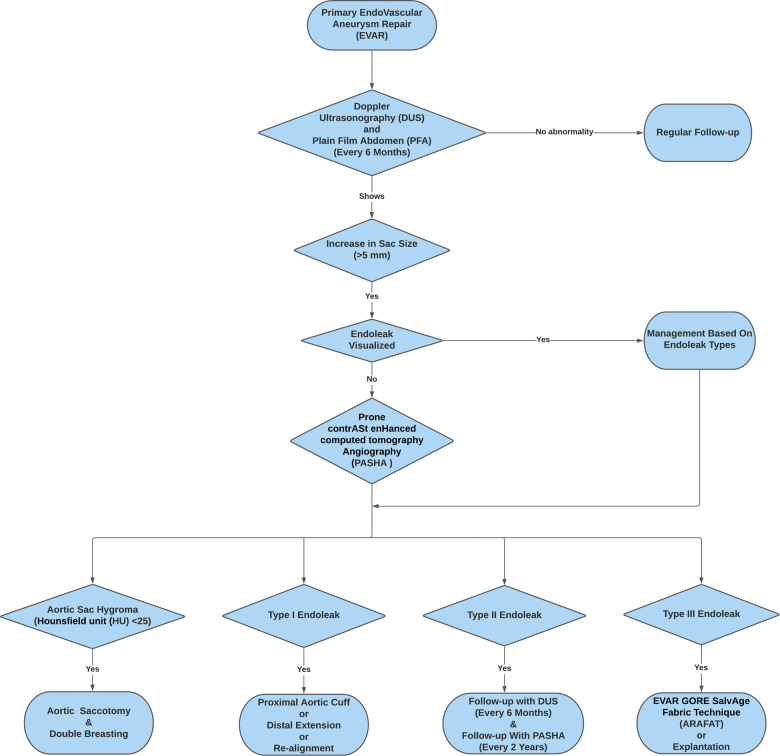
Flowchart depicting the adoption of the re-intervention strategies (EVAR GORE SalvAge FAbric Technique (ARAFAT), Double Breasting, and Explantation) following Prone ContrASt EnHanced Computed Tomography Angiography (PASHA).

We identified 136 ELs (14.95%), including 15 type I ELs, 98 type II ELs, 18 type III ELs, and 5 type IV ELs. Decisions for re-interventions were formulated at the discretion of the vascular surgery multidisciplinary team, which considered factors like the rate of sac expansion, EL types, and patient's general condition (Figure 1). All the type I ELs were managed with proximal aortic cuff and/or distal extension or chimney endovascular aneurysm repair (ChEVAR). Four (26.70%) of these type I ELs required tertiary re-intervention at 5, 7, 8 years of post-secondary intervention. Amongst the 98 type II ELs, 12 (12.24%) that were associated with aortic sac expansion had a trial of embolisation initially; however, they all had persistent sac expansion despite embolisation. We applied PASHA diagnostic technique to them, which eventually showed aortic sac hygromas or type III ELs. Out of these 12, seven underwent double breasting for aortic sac hygromas, and the remaining five had explantations due to chronic fabric fatigue with type IIIB EL within three years of re-intervention. Our isolated type II EL had a 41.84% (*n* = 41) spontaneous resolution rate, and none of them ruptured.

### Re-intervention strategies

Our practice had evolved over twenty years from diagnostics to management. Our decision-making has been influenced by several factors, including the mode of endograft failure, aneurysm sac size and patient fitness for surgery. Our ability to make informed decisions on the mode of failure evolved after we spearheaded the PASHA CTA protocol to accurately differentiate between different types of ELs and aortic sac hygromas (7). Aortic sac hygromas with the Hounsfield unit (HU) < 25 and an associated sac size greater than 7.5 cm indicate aortic aneurysm sac failure and a loss of the ability of the aortic wall to remodel (7). In cases with no defined type I, II or III ELs, and HU was less than 25, a diagnosis of expanding aortic hygroma was confirmed, and aneurysmorrhaphy was performed. In our experience, patients developing hygromas had factors, which likely contributed to the ability of fluid to exudate through the endograft material. We witnessed that the predisposing factors for aortic sac hygroma are direct oral anti-coagulant medication, administration of tissue plasminogen activator (tPA) and episodes of hypoalbuminemia (7). However, in the absence of these factors, it was considered that exudation of fluid through the endograft is likely to represent general fabric fatigue and loss of crystallinity; we considered that relining the endograft with the ARAFAT technique was more useful to exclude the failed endograft from the circulation and prevent ongoing transudation into the aortic sac. As our patients are living longer and now present 10–15 years after the index procedure, they can be late octogenarians or early nonagenarians by the time they require reintervention. This forced us to employ the ARAFAT protocol more frequently. In essence, if a patient is fit for a definitive endograft explant and open surgical repair, then this is our procedure of choice; if a patient has precedent factors that likely contributed to a hygroma and those factors are likely not to recur, then we perform an aneurysmorrhaphy; however, if the patient has an expanding sac and is unlikely to be able to tolerate such an open surgical approach, we opt for ARAFAT physically (Figure 1).

### Explantation

**Figure 2:**
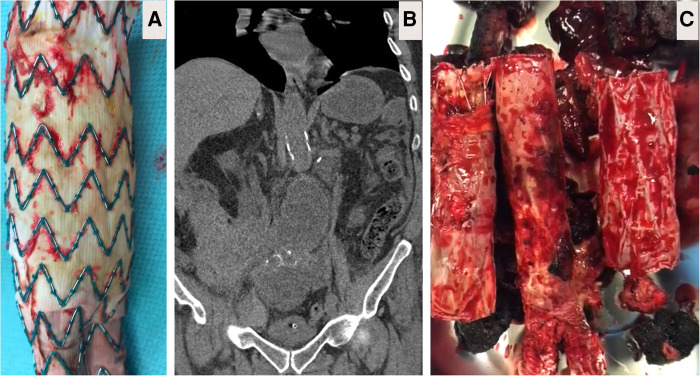
Explantation. (**A**) Body of partially explanted Cook (Bloomington, IN, USA) polyester-based endograft depicting micro-fabric pores due to chronic fatigue failure close to the allies of the stent. (**B**) Computed tomography angiography (CTA) image showing partially explanted Cook graft with the suprarenal spare springs and hooks left in-situ. (**C**) Here, patient underwent primary endovascular aneurysm repair (EVAR) with Endologix AFX (Endologix, Irvine, CA). However, this polytetrafluoroethylene (PTFE) based endograft failed due to the fracture of the endoskeleton, which acted as a hinge against the PTFE. Attempts to salvage the EVAR resulted in implantation of two proximal cuffs over six years follow-up. The patient presented with rapidly expanding 8 cm abdominal aortic aneurysm and abdominal pain, necessitating immediate total graft explantation and aorto-bi-iliac reconstruction by 16 × 8 mm silver Dacron graft (Maquet, Rastatt, Germany).

In our early series with type IIIB fabric failure, we performed explantation by infra-renal clamping after transfixing the left renal vein followed by partial endograft excision (Figures 2A,B). Once the fabric of an endograft has started to fray, it heralds the start of a more substantial issue and represents a more extensive reduction in fabric integrity and a loss of crystallinity. Placing a stitch in the fabric would be a temporary, and likely unsuccessful attempt to solve a chronic problem. In fact, stitching a frayed fabric would likely propagate further holes and fabric disintegration, causing more damage than good. Once the device has failed, it needs to be explanted or relined. During explantation, we routinely leave behind the suprarenal uncovered stents and their barbs and anastomosed the open surgical graft to it infra-renally after partial graft excision. However, the AFX endologix (Endologix Inc., Irvine, CA, USA) graft was totally removed as it depends on the iliac bifurcation for fixation and does not have barbs (Figure 2C). We routinely performed re-enforcement with polytetrafluoroethylene (PTFE) pledgets when anastomosing the silver Dacron graft to the old proximal failed aortic graft or the aortic wall. Pledgets were necessary for reinforcement as the aortic tissue was friable and required support.

However, many of our patients with type IIIB EL were between 4 and 9 years post-implantation and therefore, most were either septuagenarian or octogenarian. Open surgery and explantation are risky in these frail sarcopenic patients. Consequently, we developed the ARAFAT technique in patients at high risk for surgery, i.e., elderly patients with concurrent co-morbidities.

### Aortic sac hygroma and double-breasting

Aortic sac hygromas are sac dilatations attributed to transudation through the stent-graft fabric. They expand slowly without evidence of EL forming a phlegmon with jelly-like consistency around the endograft. They have radio-density less than 25 Hounsfield units (HU) and are most commonly associated with polyester endografts (7–11). Diagnosis of aortic sac hygromas was neither feasible nor accurate previously; however, newer imaging techniques will identify them.

**Figure 3:**
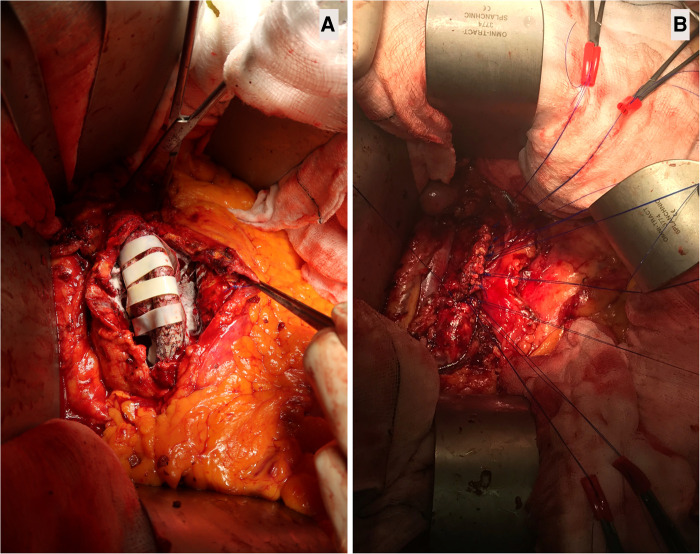
Aortic sacotomy with obliterating endo-aneurysmorrhaphy and stent-graft preservation post-EVAR. (A): Wrapping the opened aortic sac with XenoSure biological patch (LeMaitre Vascular, Inc., MA, USA) during double breasting. This 82-year-old patient had endovascular aneurysm repair (EVAR) with polytetrafluoroethylene (PTFE) GORE graft (GORE®, Flagstaff, Arizona, USA) in 2008, after which the aortic sac shrunk to 4.2 cm. However, the patient had tissue plasminogen activator (TPA) twice for acute MI in 2018, resulting in aortic sac hygroma with sac expansion to 8.9 cm. The formation of a hygroma as a consequence of ultrafiltration of blood through the stent-graft fabric led to continued sac enlargement without a detectable problem within the endograft, i.e., no structural stent-graft problem and no demonstration of EL. (B). Double breasting of the aortic sac with interrupted mattress prolene stitches to achieve hemostasis. Due to aortic sac hygroma, the patient experienced continuous sac expansion, abdominal pain, and low back pain. Computed tomography angiography (CTA) demonstrated the radiodensity of <25 Hounsfield units. The patient required aortic sacotomy, followed by wrapping the aortic ePTFE device with XenoSure biological patches and filling the post wall of the aortic sac with haemostatic powder HaemocerTM plus (Biocer Entwicklungs-Gmbh, Bayreuth, Germany), after which double breasting of the aortic sac was performed. This sealed the aortic sac and abolished the abdominal and low back pain. The aortic sac shrank to 4.5 cm during the subsequent follow-up.

We performed aortic sacotomy after the diagnosis, with the evacuation of related hygroma and/or aortic thrombus, and all bleeding lumber vessels were transfixed. Subsequently, we filled the opened aortic sac with XenoSure® biologic patches (LeMaitre Vascular, Inc., MA, USA) to induce fibrosis. We then performed aneurysmorrhaphy by double breasting and plication of the aneurysm sac over EVAR graft to prevent contact with the bowel, thereby reducing the risk of subsequent graft infection (Figures 3A, B). Wrapping and cerclage of the proximal aneurysm neck were used to prevent stent-graft migration and EVAR dislodgment. Aortic sacotomy and double breasting made it possible to confirm endotension when no visible leak was seen on the preoperative CT scan. In these cases, the aortic sac hygroma presented as dark grey organised seroma with no patent back-bleeding vessels. All hygromas were sent for culture and sensitivity, and all returned sterile.

### EVAR GORE SalvAge fabric technique (ARAFAT)

**Figure 4:**
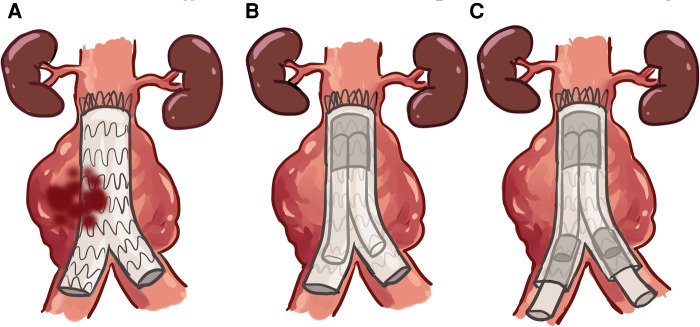
EVAR GORE SalvAge Fabric Technique (ARAFAT) is our protocol used to seal the type IIIb EL. (**A**) Microleaks at the endograft. (**B**) Oversized EXCLUDER® aortic cuff (GORE®, Flagstaff, Arizona, USA) deployed into the previously implanted stent-graft. (**C**) Simultaneous deployment of an EXCLUDER® iliac extension, as necessary, in double-barrel configuration from the main cuff.

Over the past 5 years, we utilized ARAFAT as our protocol to seal the type IIIB EL, particularly in patients at high risk for open conversion (7). ARAFAT helped us realign stent graft to seal microleaks and improve spiral arterial flow. Here, we deployed an oversized EXCLUDER® aortic cuff (GORE®, Flagstaff, Arizona, USA) into the previously implanted stent graft, followed by the simultaneous deployment of EXCLUDER® iliac extensions as necessary in double-barrel configuration from the main cuff (Figures 4A–C) (7).

### Ethical consideration

Ethical approval was obtained from the local Institutional Ethics Review Board (C.A. 1210). Data were collected from patients' records and anonymised. Utmost priority was given to maintain patients' confidentiality.

### Statistical analysis

Continuous outcomes were summarized with mean and standard deviation (normal distribution) or median and interquartile range (non-normal distribution). The categorical outcomes were summarized with percentages and proportions. For statistical significance, Chi-squared and Kruskal-Wallis tests were used. *p* < 0.05 was taken as statistically significant. Statistical analyses were conducted with Minitab (Minitab® Ltd., UK).

## Results

We had 44 patients who underwent reinterventions following primary EVAR, including 18 ARAFAT and 14 explantations for type IIIB ELs and 12 double breastings for aortic sac hygromas. The baseline characteristics of these patients are given in Table 1.

**Table 1:** Baseline characteristics of the patients.

Baseline characteristics	Re-intervention strategies				
	Overall	Types			
		ARAFAT (*N* = 18)	Double breasting (*N* = 12)	Explantation (*N* = 14)	*p*-value
Total	44	18 (40.9%)	12 (27.3%)	14 (31.8%)	–
Male, *n* (%)	33 (75%)	14 (42.4%)	10 (22.7%)	9 (20.5%)	**0** **.** **511**
Age (mean ± SD), years	79.7 ± 7.03	80 ± 6.86	81.4 ± 4.93	77.7 ± 8.63	**0** **.** **408**
Tissue Plasminogen Activator use	3	0	3	0	–
Ischemic Heart Disease	27	10	8	9	**0** **.** **803**
Coronary stenting	11	8	3	0	**0** **.** **251**
Hypercholesterolimia	33	13	9	11	**0** **.** **925**
Atrail fibrillation	9	0	4	5	**0** **.** **022**
Hypertension	37	15	9	13	**0** **.** **261**
Diabetes Mellitus	15	3	5	7	**0** **.** **129**
Renal disease	7	3	2	2	**0** **.** **583**

Abbreviations: EVAR, EndoVascular Aneurysm Repair; ARAFAT, EVAR GORE SalvAge FAbric Technique; SD, standard deviation.

The average size of the aortic aneurysm sac during the primary EVAR was 6.00 ± 1.75 cm (Table 2). Twenty-seven polyester and 17 PTFE based endo-grafts were employed in these primary procedures. The average number of stent pieces used in the primary EVAR was 3.42 ± 1.31. The proximal main body diameter was 31.6 ± 3.80 mm (right limb size: 17.6 ± 4.20 mm and left limb size 17.9 ± 4.28 mm).

**Table 2:** Endograft characteristics employed in the primary endovascular aneurysm repair.

Baseline characteristics	Re-intervention strategies				
	Overall	Types			
		ARAFAT (*N* = 18)	Double breasting (*N* = 12)	Explantation (*N* = 14)	*p*-value
AAA size (CT Scans), cm	6.00 ± 1.75	5.90 ± 1.93	6.12 ± 1.24	6.00 ± 2.05	**0** **.** **940**
Stent-graft material (PE vs PTFE)	27 (61.4%) vs 17 (38.6%)	12 vs 6	5 vs 7	10 vs 4	**0** **.** **258**
No of pieces (stents), *n*	3.42 ± 1.31	3.33 ± 1.37	3.58 ± 0.99	3.36 ± 1.55	**0** **.** **846**
Proximal aortic cuff size, mm	31.6 ± 3.80	32.3 ± 3.77	31.5 ± 4.03	30.9 ± 3.77	**0** **.** **651**
Right limb stent-graft size, mm	17.6 ± 4.20	15.8 ± 4.86	18.8 ± 4.37	18.0 ± 3.14	**0** **.** **306**
Left limb stent-graft size, mm	17.9 ± 4.28	16.3 ± 5.52	19.1 ± 4.29	18.1 ± 2.66	**0** **.** **422**

Abbreviations: AAA, abdominal aortic aneurysm; ARAFAT, EVAR GORE SalvAge FAbric Technique; CT, computerised tomography; PE, polyester; PTFE, polytetrafluoroethylene.

The mean duration of ELs identification following primary EVAR was 53.3 ± 6.82 months. The mean aortic sac expansion rate was 0.43 ± 0.25 cm per year, and the mean aortic sac size before the re-intervention was 7.51 ± 1.94 cm. Patients who underwent double breasting had the highest sac size (ARAFAT: 6.68 ± 2.13 vs. double breasting: 8.41 ± 1.72 vs. explantation: 7.52 ± 1.61 cm, *p* = 0.029). The mean duration of re-intervention following primary-EVAR was 70.2 ± 6.98 months (ARAFAT: 94.2 ± 12.5 vs. double breasting: 67.3 ± 6.78 vs. 41.8 ± 9.54 months, *p* = 0.026).

There was no difference between stent-graft materials (27 PE vs. 17 ePTFE, *p* = 0.06) on the re-intervention rate (Table 3).

**Table 3:** Stent-graft employed during the primary endovascular aneurysm repair (EVAR) and those requiring re-intervention.

Endograft types	Fabric material	Primary EVAR (total = 910)	Re-intervention (total = 44)
Zenith (Cook, Bloomington, IN, USA)	Polyester	4 (0.44%)	3 (75.00%)
Powerlink/AFX I and II (Endologix, Irvine, CA, USA)	ePTFE	110 (12.20%)	6 (5.50%)
Talent/Endurant I and II (Medtronic, Santa Rosa, CA, USA)	Polyester	330 (36.30%)	19 (5.80%)
Excluder Generation I and II (Gore Medical, Flagstaff, AZ, USA)	ePTFE	343 (37.70%)	11 (3.21%)
Incraft (Cordis, Miami Lakes, FL, USA)	Polyester	123 (13.52%)	5 (4.10%)

Abbreviation: ePTFE, expanded polytetrafluoroethylene.

All the patients had primary technical success. However, we had one sepsis-induced peri-operative mortality following explantation in a patient who had initial re-intervention for rapidly expanding aortic sac with type I EL before being referred to us. This patient also had prior embolisation of a lumbar branch, where the coil migrated to the spine resulting in paraparesis. After being referred to our centre, we performed explantation and aorto-bi-renal-bi-iliac bypass. The patient developed multiorgan failure due to sepsis and succumbed to death on the 28th postoperative day.

**Figure 5:**
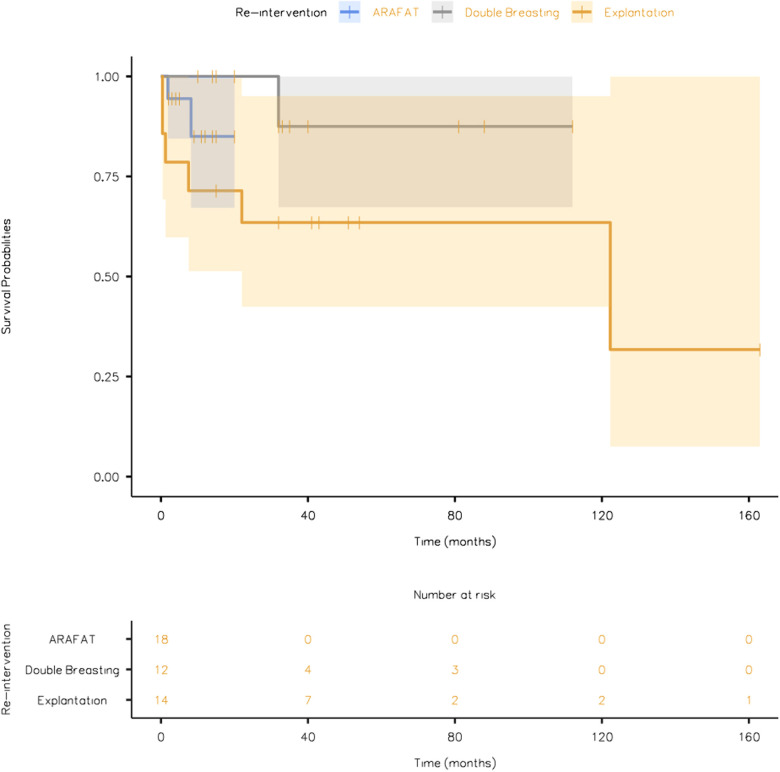
The overall survival plot of the patients undergoing different re-intervention strategies (EVAR GORE SalvAge FAbric Technique (ARAFAT), Double Breasting, and Explantation).

The overall survival plots during an average follow-up duration of 35.6 ± 6.24 months (ARAFAT: 9.00 ± 1.36 vs. double breasting: 42.5 ± 9.51 vs. explantation: 63.8 ± 14.2) is depicted in Figure 5.

## Discussion

This study aims to scrutinise our three techniques of post-EVAR re-intervention, including an EVAR graft preservation strategy and/or explantation. Significant sac expansion over a short period needs scrutiny. Stent graft explantation with subsequent replacement is the definitive management approach. Graft preservation strategies include surgical double breasting of the aortic sac or endovascular relining of the stent-graft. For patients with significant co-morbidities and an enlarged sac with a maximum diameter less than 7.5 cm, we employed ARAFAT. Our results mimic Doumenc et al. (12) finding's that explantation and endovascular management, hybrid endovascular repair and ARAFAT can be achieved in high deliberate practice volume centres with satisfactory results.

All our re-interventions had aortic sac size greater than 6 cm at the time of their primary EVAR with more than three modular stent graft components. Those that underwent double breasting had the largest sac size due to aortic hygromas (more than 7.5 cm on average) (7). Polyester was the most common stent material used in the primary EVAR amongst the re-intervention groups (*p* = 0.258).

EVAR carries a higher reintervention risk than OSR, and the risk increases with time (2). Reintervention rate could range from 20% in low-risk cases to 25% in high-risk patients (13, 14). There is a minimal divergence between first, second or third-generation aortic endograft devices (15). However, there has been an acknowledged failure rate for all major commercial endografts, necessitating late aneurysmorrhaphy and double breasting (16).

In our experience of 44 cases, we were surgically successful for a range of different endovascular scenarios and accomplished reasonable outcomes. There was no aneurysm-related mortality. However, amongst the nine mortalities over the mean follow-up duration, 60% were cancer-related, and 40% were cardiac-related.

Endograft twisting, dislodgment, kinking, outflow obstruction, and ELs are some of the complications encountered post-EVAR. However, the main reason for re-intervention is EL with endotension and sac expansion regardless of the EL types (17). Gambardella et al. (18) reported that EL is also the utmost reason for aortic sacotomy, aneurysmorrhaphy and double breasting. Also, our strategy mirrors their advice in favouring infrarenal clamping for partial explanation of failed supra renal fixated EVAR grafts.

Controversial views about EL and endotension are plentiful within the vascular literature. Studies have shown that persistent EL increases the risk of rupture (19). However, some advocates that type II ELs are protective against aortic rupture (20). Current guidelines recommend intervention in patients with continuous sac expansion (21). Variable results with trans-arterial, trans-caval, direct sac access and trans-iliac-lumbar embolization for type II EL had been reported (21). Alternative approaches such as the laparoscopic approach for type II EL has been described as tricky, even by the most proficient surgeons, due to dense periaortic inflammation (22). Minimal invasive therapies for type II ELs risk repeated interventions, with poor outcomes at five years (21, 23). In our opinion, type II EL should not be accepted as the cause of aortic sac expansion until multimodal investigations with temporal information regarding perigraft blood flow have been completed.

Our type IA ELs were challenging to solve as there was no consensus. Our cases were managed by Design Reconfigure Elongate Straighten Stiffen (DRESS) technique with proximal cuff and adjuvant ChEVAR.

We agree with the recommendation of Hinchliffe et al. (24) that in frail patients who were turned down for open repair a few years earlier, less invasive approaches with a lower threshold for endograft preservation should be considered. In this context, an aortic sacotomy with ligation of patent back-bleeding vessels and preservation of the EVAR graft can be a wise alternative to a more invasive explantation to prevent rupture in an expanding aortic sac.

Almost half of the ELs seal spontaneously during the first year; however, the likelihood of spontaneous closure decreases with time (4, 25). There is no consensus regarding how long to follow up and when to re-intervene (13–15). Only 1.97% of our patients had an endovascular reintervention before aneurysmorrhaphy, as we are highly selective in offering EVAR. The mean age of our patients who underwent re-intervention was 79.7 ± 7.03 years. In frail patients, we considered open repair only if endovascular options were impractical or exhausted. However, in cases in which the aortic sac diameter was more than 75 mm with abdominal or low back pain, we performed aneurysmorrhaphy. Any prolongation of such a critical situation could have ended in aortic sac rupture. This contradicts some authors who had one-third of all their patients subjected to endovascular manipulation before sacotomy, aneurysmorrhaphy, graft preservation and double breasting (26, 27).

Complications post-EVAR could be attributed to various factors. A head to head comparison with Endurant and Excluder grafts implanted in two groups of patients having similar anatomical characteristics demonstrated that two different types of EVAR endografts implanted in similar AAAs could provoke diverse flow properties (28). The study concluded that delineation of the hemodynamic features associated with the various commercially available EVAR grafts could further promote the personalization of treatment offered to aneurysmal patients and instigate concepts for design perfection in the future.

Published data has reported several factors associated with major adverse events post-EVAR. These factors include an aortic bifurcation with 50% calcification and diameter less than 20 mm; endograft iliac limb diameters greater than 27 mm; nitinol endograft stents; and the ratio of endograft iliac limb diameters to aortic bifurcation diameter greater than 1.4 (29). Conversely, the displacement forces in tapered iliac stent-grafts with asymmetric curvatures will impact stent-graft performance. When arterial blood pressure is on a curved stent-graft, it will generate an axially oriented force. The larger the cross-section, the more significant the force. In addition, the force generated by the flow velocity in the curvature of a vessel or a stent-graft acts in the axial direction. These two drag forces are due to the flow reaction to a change in direction due to the kinetic energy of the moving blood volume. The higher this velocity, the higher the energy. When the flow rallies into the curved wall, the kinetic energy is converted into a strong force. In smaller vessels, less than 11 mm, the force-velocity is larger than the force pressure. This breeds trepidations, particularly for stent-graft designs with 11 mm contralateral gates, which necessitates 13–14 mm contralateral docking limbs as they induce mega forces that result in major adverse events. Tapered grafts increase the axial forces applied at both ends by 50% as the flow velocity increases with a smaller diameter, increasing force-velocity. These forces increase with angulation, and implantations in angulated iliacs must be avoided to minimise migration risks. These haemodynamic forces have implications for stent-graft design for both tapered and bell-bottom geometries. A tapered graft should be outsized at both ends to augment radial and frictional force with the vessel and counterbalance velocity increase (30).

Morris et al. (31) had mirrored the above findings in the abdominal aortic endografts by analysing the Zenith (Cook, Bloomington, IN) and Endurant II (Medtronic Santa Rosa, CA) devices and documented the highest radial resistive force up to 3 N/cm. The supra-renal and infrarenal compliances were 6.9–5.1 × 104/mmHg and 4.8–5.4 × 104/mmHg. In contrast, the Fortron device (Cordis Endovascular, Santa Clara, CA) had the lowest at 0.11 N/cm. The Endurant II and Excluder devices had significantly decreased infrarenal compliance by 13%–26%. All four devices increased the pulsatile arterial energy loss (PAEL) by 44%, significantly lowering aortic wall compliance after EVAR. Choosing the most compliant devices for treating AAA minimises micro and macro-ELs and graft material fatigue and failure with later explantation with avoiding long-term cardiovascular events.

Further, long term renal outcomes with proximal aortic fixation are questionable, problematic and not yet established (32). Morris et al. (31) showed a frank dissimilarity between nitinol-based endografts with Dacron and suprarenal fixation compared to nitinol-based endografts with PTFE. Zenith (Cook Medical) and Endurant II (Medtronic) had the highest aortic stiffness (radial resistive force). Moreover, significant lower infra-renal compliance was observed in Endurant II and Excluder. Similarly, the selection of the most compliant devices will enhance aortic elastic recoil with lower post-procedural complications. The metallic endograft skeleton, whether nitinol, stainless steel or cobalt alloy, reduces aortic compliance and stiffens the aorta. This stiffness induces a mismatch in the physio-mechanical properties between the native and the stented aorta, which results in PWV intensification.

Proximal fixation devices by suprarenal barbs for wall anchoring are contemplated to lessen the risk of distal migration, perfect proximal seal, and minimise type I EL. These attributes present us with a more challenging task as the presence of suprarenal stents often leads to suprarenal or supra-visceral proximal aortic clamping. Furthermore, detachment of wall-anchoring barbs risks injury to the aortic wall and renal ostia. These challenges increase operative and aortic clamp times, explaining the high reported mortality in these patients (33, 34).

We performed a partial explantation technique by leaving the suprarenal components of stent-grafts in-situ in the absence of sepsis. This is our preferred revascularisation option to reduce the risk of intraoperative injury to the aortic wall and branch vessels. It also helps to minimise para-aortic dissection and supra visceral aortic clamp level by retaining the proximal aortic endoskeleton. We anastomose the Dacron graft enforced with pledges to the endograft components to avoid suprarenal clamping and mortality.

The incidence of late aneurysmorrhaphy and double breasting after EVAR has been reported in up to 50% of reinterventions, which is multifactorial and depends on patient selection, follow-up protocols, endograft generation and type, and expertise in both endovascular and open aortic management (35–37).

In aortic hygroma patients with aortic sac more than 8 cm with abdominal and low back pain, we perform aortic sacotomy with obliterating endo-aneurysmorrhaphy and stent-graft preservation post-EVAR.

Aortic sacotomy with obliterating endo-aneurysmorrhaphy and stent-graft preservation post-EVAR is appealing as it averts the physiologic stresses of aortic cross clamping. Our results mirror Mohapatra et al. (38) and contradict Kansal et al. (39) for their striking 43% 30-day mortality.

In our experience, we noticed no difference in patients undergoing graft preservation vs. graft explantation. However, trends indicate that the graft preservation patients' were older and a higher risk cohort.

In proceeding with graft preservation, external banding of the neck combined with ligation of all branch vessels, including inferior mesenteric artery and median sacral artery, is performed. Subsequently, the aortic sac is filled with XenoSure biological patches, and the preserved graft is wrapped with silver Dacron patches to induce fibrosis and prevent any future chance of aortic sac expansion.

Some authors advocate routine CTA follow-up post-EVAR (40, 41). However, less than 50% adhered to imaging in the EVAR-1 trial and Medicare beneficiaries after five years (42, 43). We adopted the PASHA technique over contrast-enhanced ultrasound imaging, which is operator-dependant, as PASHA is standardised, easily reproducible and can be read by everyone (7, 44, 45). We recommend lifelong follow-up every six months by DUS for both iliac arteries post explantation of aorto-bi-iliac endograft for fear of iliac degeneration and rupture, as reported by Arnaoutakis et al. (46) Postoperative CTA provides better diagnostic utility for proximal and distal neck dilatation or disconnection of the stent-graft components, which DUS could miss on regular follow-ups.

ELs are triggered by the instability of the longitudinal growth of the aorta due to the cone-shaped necks or steep angulations. The shape of the proximal portion of the stent's main body gets flattened or crushed due to the cardiovascular aortic oscillation, increasing 2–4 mm in the aortic diameter of the proximal landing zone every year. The main body migrates continuously and slowly down towards the aortic bifurcation. This creates “autologous” strut perforations with type III fabric failure and ultimately type IA that can only be salvaged by explantation or ARAFAT technique (7, 47, 48).

The ability to salvage such cases by adding FEVAR or BEVAR is deemed to fail because bridging stents are needed to provide adequate stability over time. Furthermore, new challenges have arisen concerning patency as vascular territories that are primarily unaffected are incorporated into the disease process. Moreover, devices with suprarenal fixation components preclude suitable entry to visceral and renal vessels (49). Cognisant of such findings, we strongly advocate precise and meticulous strategic primary EVAR planning.

Post-implantation syndrome (PIS) following primary procedure is also a long-term determinate of EL and micro-fabric fatigue failure. Ito et al. (50) Voûte et al. (51) and Sartipy (52) associated polyester grafts with higher postoperative pyrexia, PIS and longer in-hospital stay compared to ePTFE grafts following EVAR. Post-implantation syndrome (PIS) has been reported in up to 60% following EVAR (50, 53). Polyester triggers a higher release of inflammatory biomarkers (tumour necrosis factor-α, IL-6, IL-10, and CRP) than ePTFE *in vitro* (51, 54). However, implantation of stent-grafts made with woven polyester is not just independently associated with a stronger inflammatory response; it also results in endothelial damage. Furthermore, active fixation using penetrating hooks or barbs at the proximal aortic implantation site leads to endothelial aggression with the penetration of the foreign material. The precise balance between nickel and titanium, or even cutting and polishing the metal, will affect the antigenic properties of the nitinol (51).

It is not surprising that most of the EVAR device technology introduced over the past decade has been withdrawn from the market due to failure in sealing technology, material durability, unsupported body, stent fracture, and avulsion. Failures have also arisen when aortic device companies have iteratively lowered the profile of their devices to make them more attracted to non-surgical interventionalists (15). It is surprising that better stents have not yet been crafted after more than three decades of EVAR.

Choosing the most compliant devices for treating AAA minimises micro and macro-ELs and graft material fatigue, thereby avoiding late failure, explantation and long-term cardiovascular events. The physician involved in the decision-making should select the most appropriate EVAR graft. In general, approximately one-third of all of our EVARs were done out of instructions-for-use (IFU) with neck less than 1.5 cm and neck angulation more than 75%, and thrombus more than 3 mm at the proximal implantation site. However, the objective going forward is to recommend open surgery if the patient is physically fit. Otherwise, EVAR can be offered but cannot be undertaken outside IFU, with rare exceptions depending on patient fitness and urgency of presentation (47).

A strong policy of obeying the indications for use or abstaining from exploiting EVAR in challenging patients will alleviate the need for late aneurysmorrhaphy and reduce the need for reintervention to exceptional cases. LOCOS-1 (55) investigators documented that the broad applicability of EVAR increased late open conversion, independent of endovascular techniques innovations or advancements in-stent and fabrics materials. However, there are limited studies on the direct comparison between endografts and long-term outcomes, which need to be interpreted with caution. The majority of the available evidence-based studies represent a retrospective single centre experience with a limited subset of patients. Also, comparison among stent-grafts is restricted to devices based on personal and/or institutional preferences.

We believe that patient choice to a less invasive option is essential in the decision-making process; however, patients must be told of their alternatives during the informed consent process. Patients need to be well informed on the advantages and disadvantages of EVAR and understand that post-EVAR complication rates are still substantial.

### Study limitations

The current study is limited by its observational nature, with potential selection bias. Although the number of patients included in this study is limited, this is one of the most extensive series to date evaluating aortic sacotomy with obliterating endo-aneurysmorrhaphy and stent-graft preservation post-EVAR. We have discussed various re-intervention strategies employed in our vascular setting; however, we understand that the indications for the re-intervention are different. Furthermore, it is not feasible to make a head-on-head comparison between them. Also, most of these re-interventions were performed recently, and we lack a long-term follow-up.

## Conclusion

With the increasing popularity of EVAR, we forecast high numbers of post-procedural complications in terms of graft failure, short- and long-term hemodynamic alterations, and related morbidity and mortality. These complications necessitate the development and study of re-intervention strategies to salvage existing endograft and/or address graft-related complications.

## Data Availability

The raw data supporting the conclusions of this article will be made available by the authors, without undue reservation.
